# An observer blinded, randomized, placebo-controlled, phase I dose escalation trial to evaluate the safety and immunogenicity of an inactivated West Nile virus Vaccine, HydroVax-001, in healthy adults

**DOI:** 10.1016/j.vaccine.2018.12.026

**Published:** 2019-01-18

**Authors:** Christopher W. Woods, Ana M. Sanchez, Geeta K. Swamy, Micah T. McClain, Lynn Harrington, Debra Freeman, Elizabeth A. Poore, Dawn K. Slifka, Danae E. Poer DeRaad, Ian J. Amanna, Mark K. Slifka, Shu Cai, Venus Shahamatdar, Michael R. Wierzbicki, Cyrille Amegashie, Emmanuel B. Walter

**Affiliations:** a Duke Department of Medicine, Duke University School of Medicine, Durham, NC, USA; b Duke Human Vaccine Institute, Duke University School of Medicine, Durham, NC, USA; c Duke Department of Gynecology and Obstetrics, Duke University School of Medicine, Durham, NC, USA; d Duke Early Phase Research Unit, Duke University School of Medicine, Durham, NC, USA; e Najít Technologies Inc., Beaverton, OR, USA; f Division of Neuroscience, Oregon National Primate Research Center, Oregon Health & Science University, Beaverton, OR, USA; g National Institutes of Health, Division of Microbiology and Infectious Diseases, Bethesda, MD, USA; h Emmes Corporation, Rockville, MD, USA

**Keywords:** West Nile virus, Vaccine, Phase 1

## Abstract

**Background:**

West Nile virus (WNV) is the most common mosquito-borne infection in the United States. HydroVax-001 WNV is a hydrogen peroxide inactivated, whole virion (WNV-Kunjin strain) vaccine adjuvanted with aluminum hydroxide.

**Methods:**

We performed a phase 1, randomized, placebo-controlled, double-blind (within dosing group), dose escalation clinical trial of the HydroVax-001 WNV vaccine administered via intramuscular injection. This trial evaluated 1 mcg and 4 mcg dosages of HydroVax-001 WNV vaccine given intramuscularly on day 1 and day 29 in healthy adults. The two dosing groups of HydroVax-001 were enrolled sequentially and each group consisted of 20 individuals who received HydroVax-001 and 5 who received placebo. Safety was assessed at all study days (days 1, 2, 4 and 15 post dose 1, and days 1, 2, 4, 15, 29, 57, 180 and 365 post dose 2), and reactogenicity was assessed for 14 days after administration of each dose. Immunogenicity was measured by WNV-specific plaque reduction neutralization tests (PRNT_50_) in the presence or absence of added complement or by WNV-specific enzyme-linked immunosorbent assays (ELISA).

**Results:**

HydroVax-001 was safe and well-tolerated as there were no serious adverse events or concerning safety signals. At the 1 mcg dose, HydroVax-001 was not immunogenic by PRNT_50_ but elicited up to 41% seroconversion by WNV-specific ELISA in the per-protocol population (PP) after the second dose. At the 4 mcg dose, HydroVax-001 elicited neutralizing antibody responses in 31% of the PP following the second dose. In the presence of added complement, PRNT_50_ seroconversion rates increased to 50%, and 75% seroconversion was observed by WNV-specific ELISA.

**Conclusions:**

The HydroVax-001 WNV vaccine was found to be modestly immunogenic and welltolerated at all dose levels.

## 1. Introduction

West Nile virus (WNV) is a neurotropic flavivirus that cycles between mosquitoes and birds but can also infect humans and other vertebrate animals. WNV was first identified in the Western hemisphere in 1999 as a cluster of viral encephalitis cases in New York City [ 1 ]. Since then, WNV has spread rapidly west across the continental United States [2]. Among persons infected with WNV, the majority have subclinical infection, approximately 25% develop West Nile fever, and about 1 in 200 develops neuroinvasive disease, with manifestations including encephalitis, meningitis, and flaccid paralysis [3,4]. Neuroinvasive disease, more common among older adults, is associated with a case-fatality rate of approximately 10% [5]. Survivors often experience long-term neurological dysfunction [6] and may be at additional risk of chronic kidney disease (CKD) [7]. From 1999 through 2017, a total of 48,183 cases of WNV disease have been reported in the United States, including 22,999 cases of neuroinvasive disease and 2163 deaths [8].

At present, there is no human vaccine available for WNV infection. However, since the introduction of WNV into the United States in 1999, significant research efforts have been expended to create a viable vaccine for disease prevention in humans [9]. These efforts include live, attenuated chimeric vaccines [10–16], DNA vectored vaccines [10–19], recombinant subunit vaccines [20], and a variety of protein vaccines including chemically inactivated whole virus [21–23] and virus-like particles [24].

As an alternative to traditional formaldehyde-based vaccines, a novel hydrogen peroxide (H_2_O_2_) inactivation approach has been developed to produce a first-generation whole-virus vaccine against WNV that was tested at concentrations ranging from 1 to 40 mcg/dose [25–27]. Preliminary studies explored the use of H_2_O_2_-based inactivation of wild-type NY99 WNV for vaccine development, with vaccine antigen able to elicit robust neutralizing antibody responses in amice and protect against lethal challenge [27]. Further studies using WNV-Kunjin virus (WNV-KV), a naturally attenuated Lineage 1 WNV strain, expanded on these preliminary results [26]. Both young and aged mice immunized with H_2_O_2_-inactivated WNV-KV, formulated with aluminum hydroxide, demonstrated robust antiviral B and T cell immunity and protection in a stringent intracranial challenge model using a heterologous North American WNV strain [26]. A scaled manufacturing approach for production was established and clinical-grade vaccine, termed HydroVax-001 WNV, was produced and found to be stable, safe and immunogenic in three animal species [27]. In light of these promising results, HydroVax-001 WNV, a 3% hydrogen peroxide-inactivated, whole virion (WNV-Kunjin strain) vaccine, was administered as a two-dose intramuscular (IM) vaccine scheduled at a 28-day interval to assess safety and immunogenicity in humans. Based on pre-clinical data described above and studies of other inactivated alum-adsorbed flavivirus vaccines, the antigen doses evaluated were 1 mcg and 4 mcg.

## 2. Methods

### 2.1. Design and conduct of the clinical trial

We enrolled healthy men and non-pregnant women aged 18 through 49 years in this prospective, placebo-controlled, observer-blind, dose-escalating, Phase 1 clinical vaccine trial. The study was conducted at Duke University after review and approval by the Duke University Health System Institutional Review Board. Included subjects were required to be seronegative to West Nile virus. Subjects of childbearing potential were also required to have a negative pregnancy test on the date of screening and prior to each dose administration. For a complete listing of subject inclusion and exclusion criteria refer to clinicaltrials.gov: NCT02337868.

Following a dose escalation design, the vaccine dose increased between groups from 1 mcg HydroVax-001 (group 1) to 4 mcg HydroVax-001 (group 2). In each group, 20 subjects received vaccine and a total of 10 subjects received placebo. Within each dosing group subjects were randomized 4:1 to receive either HydroVax-001 or placebo as a two dose series delivered intramuscularly on days 1 and 29. Subjects were followed to day 365 for safety and to assess vaccine immunogenicity.

### 2.2. Study product

The HydroVax-001 vaccine is a West Nile virus-Kunjin strain, Vero cell tissue culture-derived, purified whole virion vaccine inactivated with 3% hydrogen peroxide. HydroVax-001 drug product contains 4 mcg purified whole virion WNV formulated in a volume of 0.5 mL/dose with 0.1% aluminum hydroxide and 2% sorbitol in phosphate-buffered saline [27]. Sodium Chloride Injection USP 0.9% as a sterile, nonpyrogenic, isotonic solution was used as the vaccine diluent and the placebo. The vaccine and the normal saline placebo/diluent were shipped and stored refrigerated at 2–8 °C. During storage, HydroVax-001 vials were kept in a lightproof container.

#### 2.2.1. Dose rationale

Based on animal model evaluations of HydroVax-001 [27] and studies of other inactivated alum-adsorbed flavivirus vaccines [28–30], the antigen doses evaluated in this Phase 1 trial of HydroVax-001 were 1 mcg and 4 mcg. At the time the preclinical toxicology study was performed, a 6 mcg/0.5 mL “high dose” and a 1 mcg/0.5 mL “low dose” vaccine formulation was planned for this Phase I clinical trial. However, based on further preclinical tests [27], a lower dose of the vaccine test article (4 mcg/0.5 mL) was selected as the “high dose”, to be compared with a 1 mcg/0.5 mL “low” dose.

### 2.3. Vaccine administration and masking

Study staff and investigators were not blinded to dose group. However, within dose groups, subjects, investigators, and study staff other than unblinded site research pharmacists and unblinded research nurse vaccinators were blinded as to the subject’s treatment assignment (vaccine vs. placebo). An unblinded site research pharmacist prepared the study product and an unblinded research nurse performed an IM injection of the study product into the deltoid per the randomization assignment. The pharmacist concealed the contents of the syringe by wrapping the syringe barrel with an opaque tape or other equivalent material. In addition, the subject was instructed to look away when the vaccine was administered. The unblinded site research pharmacist and unblinded research nurse were not involved in study-related assessments nor did they have subject contact for data collection following study vaccine administration. Laboratory personnel performing antibody assays were blinded to dose group and treatment assignment.

### 2.4. Data collection

The original study design included primary and secondary objectives, which comprised safety and immunogenicity outcomes, respectively.

#### 2.4.1. Safety

Safety outcomes included both solicited and unsolicited adverse events (AEs) experienced by healthy adult volunteers after vaccination with HydroVax-001 vaccine or administration of normal saline placebo.

Solicited reactogenicity events were those adverse events known to typically occur following the administration of an inactivated vaccine. To be conservative, the reactogenicity adverse events were collected on participant-completed memory aids for 14 days following each vaccination visit. Participants were also requested to measure their temperature at the same time each day or if they felt feverish and record the highest temperature for that day for 14 days following each vaccination. In addition, oral temperature, pulse and blood pressure were measured at screening, prior to each vaccination, and on day 4 and day 15 post each vaccination and performed at other study visits if clinically indicated. These events were collected in a standard, systematic format using a graded scale based on functional assessment or magnitude of reaction.

All unsolicited, non-serious AEs were documented from Study Visit 01 (day 1) through Study Visit 08 (day 57 post second vaccination). Serious AEs (SAEs) were documented from Study Visit 01 (day 1) through Study Visit 10 (day 365 post second vaccination). All SAEs were followed until resolution even if this extended beyond the study-reporting period. Resolution of an adverse event was defined as the return to pre-treatment status or stabilization of the condition with the expectation that it will remain chronic. Unsolicited AEs were assessed for relationship to study product (not related, related).

All AEs were graded for seriousness as per 21 CFR312.32 and severity (mild, moderate, or severe).

#### 2.4.2. Laboratory safety data

Blood samples for safety laboratory assessments were collected at screening, at second vaccination, and at 4 and 15 days following each vaccination. Safety laboratory evaluations included hematology tests (hemoglobin, white blood cell count and platelet count) and blood chemistry tests (creatinine, blood urea nitrogen, glucose, potassium, alanine aminotransferase, aspartate aminotransferase, and total bilirubin). Urine was tested by dipstick at screening for glucose and protein and on day 29 after the first vaccination and day 15 after the second vaccination for glucose, protein, blood, and leukocyte esterase.

#### 2.4.3. WNV viremia

Four days after each vaccination, blood samples were tested for WNV viremia using a standard virus plaque assay on Vero cells [27]. Briefly, undiluted serum samples were adsorbed in duplicate onto confluent Vero cell monolayers in 6-well tissue culture plates (0.20 mL per well, or 0.40 mL per sample) for 1 h at 37 °C/5%CO_2_. Serum samples were aspirated and monolayers overlaid with 3 mL of 0.5% agar in 1X EMEM supplemented with 2.5% FBS, 2 mM L -glutamine and 1 × penicillin/streptomycin, returned to 37 °C/5%CO_2_ and incubated for 2 days. Wells were overlaid with 1 mL of 0.015% neutral red in 1% agar, with plaques enumerated the following day. Each experiment included a medium-only plate, as well as a virus-only plate with approximately 50 plaque forming units (PFU) per well. If no plaques were visible for a sample, it was considered as having <2.5 PFU/mL (<1 PFU per 0.40 mL of sample tested).

#### 2.4.4. Immunogenicity

##### 2.4.4.1. Plaque-reduction neutralization titer assay

The *a priori* immunogenicity objectives included assessing WNV-specific plaque reduction neutralization test (PRNT_50_) responses 29 days after a first dose and 57 days after a second dose of HydroVax-001 WNV vaccine given at doses of 1 mcg and 4 mcg. A complement-enhanced PRNT_50_ was later included in the study. PRNT_50_ assays were conducted as previously described [26,27]. Complement-enhanced PRNT50 assays were performed similar to prior descriptions [31] using human C1q (50 mcg/mL, Complement Technology, Inc, Tyler, TX). Seroconversion was defined as a 4-fold or greater increase in neutralizing antibody titer from baseline (prior to first vaccination). Geometric mean PRNT_50_ titers were determined at days 15 and 29 after first vaccination and at days 15, 29, 57, 180, and 365 days after the second vaccination. The limit of detection in the neutralizing assay is a titer of <10. For the purposes of determining seroconversion rates and GMT, antibody titers of <10 have been assumed to be 5 (one dilution step below the assay limit of detection). Therefore, seroconversion is defined as a postvaccination titer of ≥20.

##### 2.4.4.2. ELISA

The protocol was amended to include an exploratory immunogenicity objective to assess WNV-specific ELISA responses. For the ELISA analyses, this assay is performed by testing serial dilutions of serum for reactivity against WNV in an ELISA format as previously described [25]. The values reported represent the reciprocal of the last serum dilution in which a sample scored positive in the assay. The exact values are calculated via regression analysis (serum dilution vs. ELISA signal). All serum dilutions were started at a 1:30 serum dilution.

Based on previous experience with the assay and human serum samples, a conservative “limit of detection” cut-off of 200 ELISA units was utilized. This cut-off has been used in previous studies for a range of other viruses [32]. If a subject’s baseline ELISA value was <200 and their follow-up visit ELISA value was >200, this was considered seroconversion. Alternatively, for subjects with a baseline ELISA value >200, the subject needed to demonstrate a fourfold rise at follow-up for seroconversion.

### 2.5. Statistical analysis

This study was exploratory, designed to estimate event rates and patterns of immune responses rather than to test formal statistical hypotheses. The analysis populations including the safety population (SP) included all eligible subjects who received at least one dose of study vaccine. The modified intent-to-treat (ITT) population includes all eligible subjects who received at least one dose of study vaccine and contributed both pre- and at least one post-study vaccination blood samples for testing for which valid results were reported. The per protocol (PP) population excludes subjects who did not receive both doses of study vaccine or who had major protocol deviations, such as receipt of non-study vaccines during the time frame prohibited by the protocol or receipt of the second study vaccination substantially out of window.

#### 2.5.1. Safety

Safety was assessed at all study days (days 1, 2, 4 and 15 post dose 1, and days 2, 4, 15, 29, 57, 180 and 365 post dose 2), and reactogenicity was assessed for 14 days after administration of each dose. Vaccine dose groups were compared for baseline characteristics including demographics and laboratory measurements using descriptive statistics. The analyses of safety data were primarily descriptive.

Solicited AEs and laboratory toxicities were analyzed by taking the most severe response over the follow-up period, dichotomizing into a binary variable (none versus mild, moderate, or severe) and using exact confidence intervals to summarize the reactogenicity and toxicity rates.

Unsolicited AEs were coded by the Medical Dictionary for Regulatory Activities (MedDRA) for preferred term (PT) and system organ class (SOC). The rate and exact 95% confidence intervals of related AEs in aggregate, and by MedDRA categories, were computed.

#### 2.5.2. Immunogenicity

Immunogenicity analyses were performed using both the modified ITT population and the PP population. Rates of seroconversion for PRNT50 titer, complement-enhanced PRNT_50_ titer, and ELISA titer were summarized by tabulating the frequency of positive responses by treatment group at day 29 after first vaccination, day 57 after second vaccination in addition to other serologic time points. Response rates for each treatment group are presented with their corresponding 95% confidence interval estimates at each time point.

The geometric mean PRNT_50_ titer, complement-enhanced PRNT_50_ titer, and ELISA titer (GMT) and associated exact 95% confidence interval were calculated by treatment group at baseline, days 15 and 29 after the first vaccination and days 15, 29, 57, 180, and 365 after the second vaccination. For each study day, the number of subjects in the immunogenicity populations with available immunogenicity data at the particular study day was used in calculations. The geometric mean fold rise (GMFR) for each immunogenicity assay and associated exact 95% confidence interval were presented by treatment group at days 15 and 29 after the first vaccination and days 15, 29, 57, 180 and 365 after the second vaccination.

## 3. Results

### 3.1. Study participants

**Fig. 1.**
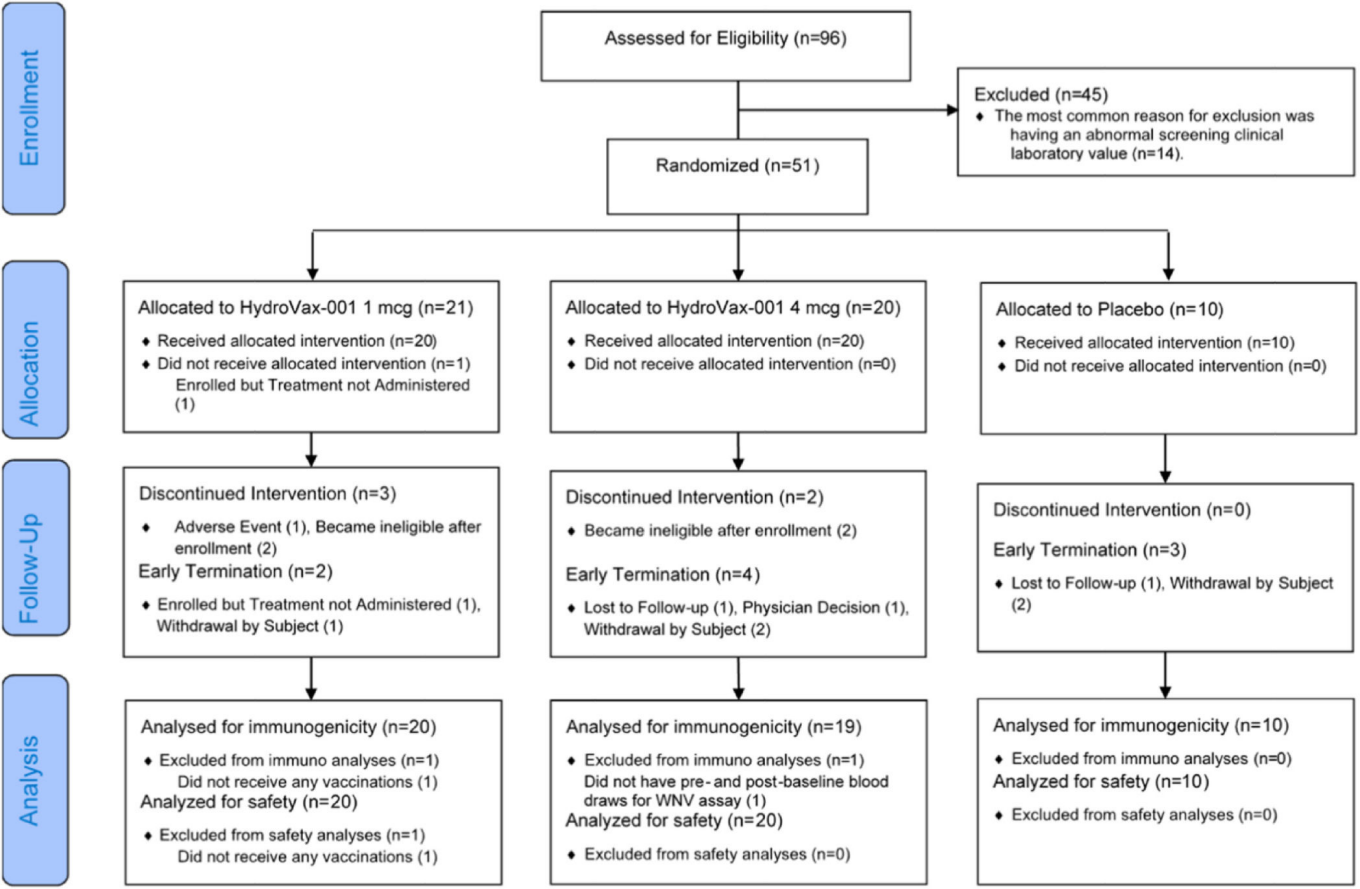
Consort diagram.

A total of 96 subjects were screened to enroll 51 healthy male and non-pregnant females into this clinical trial between March 31, 2015 and November 20, 2015. Of the total of 51 subjects enrolled, 50 (98%) received the first vaccination, and 43 (84%) received the second vaccination with HydroVax-001 or placebo. One subject was randomized and enrolled but did not receive the first vaccination and was subsequently replaced. The subjects excluded and analysis populations are illustrated and described in a CONSORT flow diagram, Fig. 1 and Supplemental Table 1. In the HydroVax-001 1 mcg group, one subject was excluded from the modified ITT and PP populations for not receiving any vaccinations, and three subjects were excluded from the PP population for not receiving both vaccinations. In the HydroVax-001 4 mcg group, one subject was excluded from the modified ITT analysis population for not having pre- and post-baseline blood draws for the WNV assay, and four subjects were excluded from the PP population for not receiving both vaccinations (including the subject without pre- and post-baseline blood draws for the WNV assay). Baseline participant demographics, age, and BMI for the 51 participants are shown in Table 1. Overall, most subjects were female (73%), non-Hispanic (96%), and white (69%). The mean age was 31.3 years and ranged from 18 years to 48 years.

### 3.2. Safety

#### 3.2.1. Solicited events

**Fig. 2.**
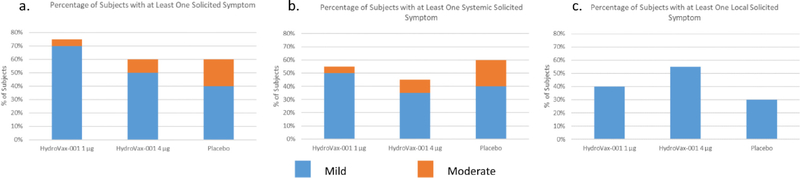
Summary of solicited events observed after any dose. The percentage of subjects that experienced at least one solicited event (a), systemic solicited event (b), and local solicited event (c) after any dose are shown by severity for Hydrovax-001 (1 μg and 4 μg, N = 20) and placebo (N = 10). No severe events were observed.

A total of 33 subjects (66%) reported solicited events after either vaccination with HydroVax-001 WNV or placebo, with 26 subjects (52%) reporting at least one systemic symptom and 22 subjects (44%) reporting at least one local symptom. The breakdown according to treatment group of adverse events by severity for at least one solicited symptom, at least one systemic solicited symptom or at least one local solicited symptom is shown in Fig. 2. A complete listing of all solicited adverse events following placebo or either vaccine dose can be found in Supplemental Tables 2–4. The most commonly reported systemic symptoms were fatigue and headache, which were distributed similarly among all groups. Fatigue was experienced at a rate of 35% (7 subjects) in the 1 mcg group, 30% (6 subjects) in the 4 mcg group, and 40% (4 subjects) in the placebo group. Reported rates of headache were similar with 30% (6 subjects) in the 1 mcg group, 20% (4 subjects) in the 4 mcg group, and 50% (5 subjects) in the placebo group. The most commonly reported local symptom was tenderness, including 40% (8 subjects) in the 1 mcg group, 45% (9 subjects) in the 4 mcg group, and 30% (3 subjects) in the placebo group. There were no Grade 3 (Severe) systemic events reported after either vaccination. There were no Grade 2 (Moderate) or 3 (Severe) local events reported after either vaccination with HydroVax-001 or placebo.

Solicited events following Doses 1 and 2 are summarized in Supplemental Tables 3 and 4, respectively. Following Dose 1, 29 (58%) subjects reported any solicited symptom with 22 (44%) reporting a systemic symptom: 10 (50%) in the 1 mcg group, 6 (30%) in the 4 mcg group, and 6 (60%) in the placebo group. Five subjects (10%) reported Grade 2 systemic events after the first vaccination: one subject (5%) in the HydroVax-001 1 mcg group reported muscle pain, two subjects (10%) in the HydroVax-001 4 mcg group reported nausea and one of those also reported fatigue, and two subjects (20%) in the placebo group reported headache and one of those also reported fatigue. Following Dose 2, 22 (51%) subjects reported any solicited symptom with 14 (33%) reporting a systemic symptom: 6 (35%) in the 1 mcg group, 4 (25%) in the 4 mcg group, and 4 (40%) in the placebo group. The most frequently reported systemic events were headache (9 subjects, 21%) and fatigue (6 subjects, 14%). There were no reported Grade 2 or Grade 3 systemic events following the second vaccination with HydroVax-001 or placebo. In total, solicited symptoms were similar across the treatment groups.

#### 3.2.2. Unsolicited events

A total of 56 non-serious unsolicited adverse events were reported among 29 subjects during the period of enrollment through day 57 following the second vaccination, including among 10 subjects (50%) in the 1 mcg group, 11 subjects (55%) in the 4 mcg group, and 8 subjects (80%) in the placebo group (data not shown). None of the unsolicited adverse events were considered severe; 11 events (20%) were considered moderate; and 45 events (80%) were considered mild. Of the total unsolicited events, 11 (among 8 subjects) were considered related to the study product. In this first-in-person clinical study, relatedness was assumed if there was a known temporal relationship between administration of the study product and the adverse event, and no alternate etiology was identified. None of the related unsolicited adverse events were considered severe; 2 events were considered moderate (1 in the 1 mcg group and 1 in the 4 mcg group); and 9 events were considered mild (3 in 1 mcg group, 5 in 4 mcg group 1 in placebo group). When comparing related events by study group, 4 events in 2 subjects (10%) were observed in the 1 mcg group, 6 events among 5 subjects (25%) were reported in the 4 mcg group and 1 event in 1 subject (10%) was described in the placebo group. The most common System Organ Classes among related non-serious adverse events were Gastrointestinal disorders (2 events in 1 mcg group, 2 events in 4 mcg group), of which Diarrhea was the most common (1 event in 1 mcg group, 1 event in 4 mcg group). There were no discernible differences between treatment groups for both all and related unsolicited adverse events. There were no serious adverse events reported at any time during the study.

#### 3.2.3. Clinical laboratory evaluations

Overall, seven subjects (14%) experienced abnormal hematology results considered related to study product including 2 subjects (10%) among the 1 mcg group, 4 in the 4 mcg group (20%) and 1 in the placebo group (10%). Three subjects (6%), (1 in 1 mcg group, 2 in 4 mcg group) had mild hemoglobin decreases and 3 (6%), (1 in 1 mcg group, 2 in 4 mcg group) experienced a mild WBC increase. One subject (2%) in the placebo group experienced a mild platelet decrease. No severe abnormal hematology results were reported. Seventeen subjects (34%) (5 in 1 mcg group, 8 in 4 mcg group, 4 in placebo group) experienced an abnormal chemistry result considered related to dose administration by the blinded investigator including two with severe abnormalities. One subject had a severe increase (5.8 mmol/L) in potassium on day 4 following a second mock vaccination in the placebo group. The potassium value was repeated four days later at a supplemental visit and was reported as 5.2 mmol/L (mild). The potassium value normalized to 4.5 mmol/L at the next scheduled protocol visit, 7 days after the supplemental visit. A different subject experienced a severe reduction in glucose to 54 mg/dL on day 29 prior to the second vaccination with 4 mcg. This subject had reported a mild glucose decrease of 65 mg/dL at baseline and glucose returned to normal levels by the next visit on day 4 post second vaccination. No laboratory abnormalities were associated with symptoms.

#### 3.2.4. Viremia

Blood samples were obtained on day 4 after each vaccination with HydroVax-001 or placebo for WNV viremia testing using a standard plaque assay. There were no positive viremia results for any subject at either time point.

### 3.3. Immunogenicity results

Seroresponses were determined using the plaque reduction neutralization test 50% reduction (PRNT_50_), a complement-enhanced PRNT_50_ assay, and a WNV-specific enzyme linked immunosorbent assay (ELISA).

#### 3.3.1. PRNT_50_ and complement-enhanced PRNT_50_ seroresponses

All subjects enrolled were seronegative at baseline as determined by the PRNT_50_ assay. At the 1 mcg dose, Hydrovax-001 did not elicit PRNT50 seroconversion following either the first or the second dose of Hydrovax-001 (Table 2 for PP and Supplemental Table 5 for modified ITT Population). As expected, none of the participants in the placebo group seroconverted to WNV during the course of the study (data not shown). At the 4 mcg dose, Hydrovax-001 did not induce neutralizing antibody titers following a single dose, but reached 31% seroconversion within 15 days after a second dose of vaccine in the PP population with antibody responses waning at days 180 and 365 post Dose 2 (Table 2). Results in the modified ITT analysis were similar with a slightly lower seroconversion rate of 28% reported at days 15 and 29 post Dose 2 (Supplemental Table 5).

The complement-enhanced PRNT_50_ seroresponses paralleled the PRNT_50_. For the 1 mcg dose of Hydrovax-001, neutralizing antibody responses were below the limit of detection (Table 3) and seroconversion was not observed among any placebo recipients (data not shown). Following a single 4 mcg dose in the PP population, one subject (6%) seroconverted at days 15 and 29 and 15 days following a second dose of vaccine, seroconversion rates increased to 50% before declining thereafter (Table 3). Results from the modified ITT analysis were slightly lower, reaching a peak seroconversion rate of 44% at 15 days following the second dose and declining to 12–19% at later time points (Supplemental Table 6).

As expected, baseline GMTs as assessed by both the PRNT_50_ and complement-enhanced PRNT_50_ were negative. Though limited in magnitude, a non-significant rise in GMT in the HydroVax-001 1 mcg group was observed at day 29 post Dose 2, but only with the PRNT50 assay and not the complement-enhanced PRNT_50_ assay. Significant increases in GMTs in the HydroVax-001 4 mcg group, as detected by both assays, occurred at days 15 and 29 post Dose 2 but returned close to baseline by day 57 post Dose 2 (Tables 2 and 3). Results in the modified ITT analysis were similar (Supplemental Tables 5 and 6).

#### 3.3.2. ELISA seroresponses

Exploratory immunogenicity results as assessed by ELISA were more robust than those as assessed by PRNT_50_ and complement-enhanced PRNT50 assays (Table 4 for PP Population and Supplemental Table 7 for modified ITT Population). At the 1 mcg dose, Hydro-Vax-001 induced seroconversion in 18% of subjects at day 15 following a first dose increasing to 41% of the subjects responding by 15 days following a second dose. At the 4 mcg dose, Hydro- Vax-001 induced seroconversion in 13% of subjects at day 29 following a first dose and this increased to 75% of subjects seroconverting by day 29 following a second dose before declining thereafter with kinetics similar to the PRNT_50_ assays. ELISA results for both PP and modified ITT analysis populations were similar (Table 4 and Supplemental Table 7). Increases in ELISA GMTs over baseline were not observed in the Hydro-Vax-001 1 mcg group. Small increases in ELISA GMTs over baseline were observed in the Hydro-Vax-001 4 mcg group at days 15 and 29 post Dose 1 with further increases noted at days 15, 29 and 57 post Dose 2, with GMTs returning to near baseline at days 180 and 365 following dose two.

## 4. Discussion

This Phase 1 dose escalation study of HydroVax-001 demonstrated that HydroVax-001 was safe and well tolerated as there were no concerning safety signals. At the 1 mcg dose, HydroVax- 001 was not immunogenic by PRNT_50_ seroconversion but we observed up to 41% seroconversion based on WNV-specific ELISA. At the 4 mcg dose, HydroVax-001 was modestly immunogenic following a second dose of vaccine with 31% to 50% seroconversion identified by standard PRNT_50_ or by a complement-enhanced PRNT50, respectively. Seroconversion at the 4 mcg dose reached a peak of 75% among the per-protocol population by WNV-specific ELISA before declining substantially at later time points.

Previous studies have demonstrated the role of complement in the development of protective WNV antibody responses and demonstrated that addition of complement enhances neutralization activity [31]. Overall, the complement-enhanced PRNT_50_ seroresponses paralleled the PRNT_50_ responses and at the 4 mcg dose, HydroVax-001 WNV vaccination was more immunogenic by complement-enhanced PRNT_50_ ( 50% seroconversion) compared to standard PRNT_50_ assays (31% seroconversion) among the PP population.

In additional exploratory analyses, seroresponses were assessed using a WNV-specific ELISA assay. Exploratory immunogenicity results assessed by ELISA were more robust than those as assessed by PRNT_50_ and the complement-enhanced PRNT_50_ assays. At the 1 mcg dose, HydroVax-001 induced a seroresponse in 18% of PP subjects at day 15 following a first dose with up to 41% responding by day 15 following a second dose. At the 4 mcg dose, HydroVax-001 induced a seroresponse in 13% of subjects at day 29 following a first dose with up to 75% of subjects seroconverting by day 29 following a second dose before declining thereafter.

Although a number of preclinical WNV vaccine approaches have been developed, few have proceeded to human clinical trials [9]. A 3-dose WNV DNA vaccine, administered at 4 mg per dose, resulted in an average PRNT_50_ = 50 (range 16–128) one month after the third immunization [17,19]. Two chimeric live-attenuated vaccines, based on either dengue serotype 4 (rWN/DEN4Δ30) or YFV-17D (ChimeriVax-WN) backbones expressing WNV prM/Env proteins, have been tested in humans [10–13]. The rWN/DEN4Δ30 vaccine demonstrated primary seroconversion rates of 55–75% depending on the vaccine dosage and geometric mean neutralizing antibody titers that peaked between 28 and 42 days after vaccination before declining to PRNT_60_ = 15–76 by 180 days after vaccination [10]. Booster vaccination at day 180 increased seroconversion rates to 89%, although geometric mean PRNT_60_ levels reached a peak of only 57 (range, 17–134). The live-attenuated ChimeriVax-WN vaccine induced a robust antiviral antibody response with >96% seroconversion, but by 12 months postvaccination the PRNT50 levels had declined to 58 for subjects between 41 and 64 years of age (PRNT_50_= 116 for all subjects). It is unclear how long antiviral immunity after ChimeriVax-WN might be maintained [33].

Inactivated whole virus vaccines remain an important class of vaccine candidates for WNV prevention. The first licensed veterinary WNV vaccine was based on this approach, using a formalin-inactivated crude viral harvest of WNV-NY99, formulated with a squalene-based adjuvant, to induce protective immunity in horses as well as other animal models [25,26], and additional studies have provided evidence of efficacy for formalin-inactivation as a potential WNV vaccine [34,35]. From a clinical perspective, one concern for these formalin-inactivated WNV vaccine candidates is that they are based on pathogenic strains of WNV. The use of highly pathogenic strains of virus for inactivated vaccines creates logistical issues associated with the as a potential WNV vaccine [34,35]. From a clinical perspective, one concern for these formalin-inactivated WNV vaccine candidates is that they are handling of BSL3 pathogens during large-scale cGMP manufacturing, in addition to safety concerns if complete inactivation is not achieved. As an alternative to traditional formaldehyde-based vaccines, this novel hydrogen peroxide (H_2_O_2_) inactivation approach has been developed to produce a first-generation whole-virus vaccine against WNV [25–27] using a naturally attenuated (BSL2) Kunjin strain of West Nile virus (WNV-KV). Hence, there are potential advantages with respect to vaccine manufacturing and vaccine safety.

Similar to our clinical study, other groups using inactivated whole virus vaccines have found it challenging to elicit durable neutralizing antibody responses against WNV [36]. In a Phase I/II dose escalation study involving 320 subjects, WNV-specific neutralizing antibody titers were low after primary vaccination but appeared to increase to geometric mean titers of between 50 and 75 by day 56 (i.e., 28 days after 2nd dose) before declining to near baseline levels by day 180 post-vaccination [36]. A range of vaccine doses (1.25, 2.5, 5, and 10 mcg/dose) was tested and no major improvement in antibody persistence was observed even after vaccination with a maximum dose of 10 mcg of vaccine. At day 180, the investigators performed a third vaccination and this resulted in antibody titers that were greatly improved over the first or second dose of vaccine and suggests that our vaccine approach could likewise be improved if a third dose of vaccine was administered on a similar vaccination schedule. In addition, a recent advance in peroxide-based inactivation technology represents another approach to improving future WNV vaccine design [37]. The first published use of 3% peroxide for virus inactivation/vaccine development was described in 2012 [25] and several improvements in oxidation-based virus inactivation have been developed since that time. In a recent study [37], Quintel et al., have developed a site-directed, Fenton-type oxidation approach that leads to more efficient virus inactivation with improved retention of neutralizing epitopes and a greater than 100-fold improvement in vaccine-mediated WNV-specific neutralizing antibody responses in mice. If a similar improvement in immunogenicity is observed in human subjects, then it is possible that higher and potentially more durable antibody responses may be attained. This represents an exciting area for further investigation and may be applicable to not only improved vaccine development against WNV, but may also be suitable for improving vaccines against other clinically important flaviviruses including yellow fever, dengue, and Zika viruses.

## Supplementary Material

1

## Figures and Tables

**Table 1:** Baseline demographics and characteristics of study participants.

		HydroVax-001 1 mcg (N = 21)		HydroVax-001 4 mcg (N = 20)		Placebo (N = 10)		All subjects (N = 51)	
Demographic category	Characteristic	n	%	n	%	n	%	n	%

Sex	Female	17	81	13	65	7	70	37	73
	Male	4	19	7	35	3	30	14	27
Ethnicity	Hispanic or Latino	2	10	0	0	0	0	2	4
	Not Hispanic or Latino	19	90	20	100	10	100	49	96
	Not reported	0	0	0	0	0	0	0	0
Race	Asian	0	0	3	15	1	10	4	8
	Black or African American	3	14	4	20	2	20	9	18
	White	18	86	10	50	7	70	35	69
	Multi-racial	0	0	3	15	0	0	3	6
Demographic/variable	Statistic	HydroVax-001 1 mcg (N = 21)		HydroVax-001 4 mcg (N = 20)		Placebo (N = 10)		All subjects (N = 51)	

Age	Mean	32.7		30.6		29.6		31.3	
	Standard deviation	9.5		8.2		6.8		8.5	
	Median	30.0		27.5		26.5		28.0	
	Minimum	18		21		23		18	
	Maximum	48		48		41		48	
BMI	Mean	26.0		25.0		24.9		25.4	
	Standard deviation	4.4		4.2		4.1		4.2	
	Median	25.2		23.9		24.0		24.3	
	Minimum	19.2		19.0		21.1		19.0	
	Maximum	33.2		33.3		32.2		33.3	

**Table 2:** Standard PRNT_50_ Geometric Mean Titer (GMT) Results, Geometric Mean Fold Rise (GMFR) and Seroresponse (4-Fold Rise) by Study Day and Treatment Group, Per Protocol Population.

	HydroVax-001 1 mcg							HydroVax-001 4 mcg						
Visit	N	4-fold rise n (%)	95% CI	GMT	95% CI	GMFR	95% CI	N	4-fold rise n (%)	95% CI	GMT	95% CI	GMFR	95% CI
Baseline	17	–	–	5.0	–	–	–	16	–	–	5.0	–	–	–
Day 15 Post Dose 1	17	0 (0)	0, 20	5.0	–	1.0	–	16	0 (0)	0, 21	5.0	–	1.0	–
Day 29 Post Dose 1	17	0 (0)	0, 20	5.0	–	1.0	–	16	0 (0)	0, 21	5.0	–	1.0	–
Day 15 Post Dose 2	17	0 (0)	0, 20	5.0	–	1.0	–	16	5 (31)	11, 59	9.8	6.2, 15.4	2.0	1.2, 3.1
Day 29 Post Dose 2	17	0 (0)	0, 20	5.2	4.8, 5.7	1.0	1.0, 1.1	16	5 (31)	11, 59	10.2	5.5, 18.9	2.0	1.1, 3.8
Day 57 Post Dose 2	17	0 (0)	0, 20	5.0	–	1.0	–	15	2 (13)	2, 40	6.4	4.8, 8.6	1.3	1.0, 1.7
Day 180 Post Dose 2	17	0 (0)	0.0, 20	5.0	–	1.0	–	15	0 (0)	0, 22	5.0	–	1.0	–
Day 365 Post Dose 2	16	0 (0)	0, 21	5.0	–	1.0	–	15	0 (0)	0, 22	5.2	4.7, 5.8	1.1	1.0, 1.2

**Table 3:** Complement-Enhanced PRNT_50_ Geometric Mean Titer (GMT) Results, Geometric Mean Fold Rise (GMFR) and Seroresponse (4-Fold Rise) by Study Day and Treatment Group, Per Protocol Population.

	HydroVax-001 1 mcg							HydroVax-001 4 mcg						
Visit	N	4-fold rise n (%)	95% CI	GMT	95% CI	GMFR	95% CI	N	4-fold rise n (%)	95% CI	GMT	95% CI	GMFR	95% CI
Baseline	17	–	–	5	–	–	–	16	–	–	5	–	–	–
Day 15 Post Dose 1	17	0 (0)	0, 20	5.0	–	1.0	–	16	1 (6)	0, 30	5.7	4.3, 7.5	1.1	0.9,1.5
Day 29 Post Dose 1	17	0 (0)	0, 20	5.0	–	1.0	–	16	1 (6)	0, 30	5.7	4.3, 7.5	1.1	0.9,1.5
Day 15 Post Dose 2	17	0 (0)	0, 20	5.0	–	1.0	–	16	8 (50)	25, 75	13.2	7.8, 22.5	2.7	1.6,4.5
Day 29 Post Dose 2	17	0 (0)	0, 20	5.0	–	1.0	–	16	6 (38)	15, 65	11.4	7.1, 18.2	2.3	1.4,3.6
Day 57 Post Dose 2	17	0 (0)	0, 20	5.0	–	1.0	–	15	2 (13)	2, 40	6.6	4.8, 9.1	1.3	1.0,1.8
Day 180 Post Dose 2	17	0 (0)	0, 20	5.0	–	1.0	–	15	3 (20)	4, 48	6.6	4.8, 9.1	1.3	1.0,1.8
Day 365 Post Dose 2	16	0 (0)	0, 21	5.0	–	1.0	–	15	2 (13)	2, 40	6.2	4.5, 8.3	1.2	0.9,1.7

**Table 4:** Seroresponse^a^, ELISA Geometric Mean Titer (GMT) Results, and Geometric Mean Fold Rise (GMFR) by Study Day and Treatment Group, Per Protocol Population.

	HydroVax-001 1 mcg							HydroVax-001 4 mcg						
Visit	N	Seroconversion n (%)	95% CI	GMT	95% CI	GMFR	95% CI	N	Seroconversion n (%)	95% CI	GMT	95% CI	GMFR	95% CI
Baseline	17	–	–	65.0	39.5, 107.0	–	–	16	–	–	42.4	34.3, 52.4	–	–
Day 15 Post Dose 1	17	3 (18)	4, 43	121.7	75.4, 196.6	1.9	1.4, 2.5	16	1 (6)	0, 30	116.1	90.6, 148.7	2.7	2.1, 3.6
Day 29 Post Dose 1	17	1 (6)	0, 29	118.4	72.4, 193.8	1.8	1.4, 2.4	16	2 (13)	2, 38	123.7	98.0, 156.0	2.9	2.2, 3.9
Day 15 Post Dose 2	17	7 (41)	18, 67	155.0	94.3, 254.9	2.4	1.6, 3.5	16	12 (75)	48, 93	479.8	287.5, 800.8	11.3	6.7, 19.0
Day 29 Post Dose 2	17	5 (29)	10, 56	152.0	89.7, 257.4	2.3	1.6, 3.5	16	12 (75)	48, 93	468.2	294.6, 744.2	11.1	7.0, 17.5
Day 57 Post Dose 2	17	5 (29)	10, 56	148.0	93.0, 235.8	2.3	1.7, 3.1	15	10 (67)	38, 88	300.6	201.7, 448.1	7.0	4.5, 10.9
Day 180 Post Dose 2	16	0 (0)	0, 21	63.0	36.6, 108.3	1.0	0.8, 1.2	15	1 (7)	0, 32	64.7	47.4, 88.2	1.5	1.1, 2.0
Day 365 Post Dose 2	16	1 (6)	0, 30	70.3	40.1, 123.3	1.0	0.8, 1.3	15	1 (7)	0, 32	61.4	44.6, 84.4	1.4	1.1, 1.8

a If a subject’s baseline ELISA value was <200 and their follow-up visit ELISA value was >200, this was considered seroconversion. Alternatively, for subjects with a baseline ELISA value >200, the subject needed to demonstrate a four-fold rise at follow-up for seroconversion.
