# Supplementary material for: An observer blinded, randomized, placebo-controlled, phase I dose escalation trial to evaluate the safety and immunogenicity of an inactivated West Nile virus Vaccine, HydroVax-001, in healthy adults
Source: Vaccine. Author manuscript; Available in PMC 2019 Jul 19. (PMC6640644; doi:10.1016/j.vaccine.2018.12.026)
Supplement: 1 [file NIHMS1022358-supplement-1.pdf]

Supplemental Table 1. Analysis Populations by Treatment Group

		HydroVax-001 1 mcg (N=21)		HydroVax-001 4 mcg (N=20)		Placebo (N=10)		All Subjects (N=51)	
**Analysis Population**	**Reason Subjects Excluded**	**n**	**%**	**n**	**%**	**n**	**%**	**n**	**%**
Safety Analysis Population	Any Reason	1	5	0	0	0	0	1	2
	Did not receive any vaccinations	1	5	0	0	0	0	1	2
Modified Intent-to-Treat Analysis Population	Any Reason	1	5	1	5	0	0	2	4
	Did not have pre- and post-baseline blood draws for WNV assay	0	0	1	5	0	0	1	2
	Did not receive any vaccinations	1	5	0	0	0	0	1	2
Per-Protocol Analysis Population	Any Reason	4	19	4	20	0	0	8	16
	Did not receive any vaccinations	1	5	0	0	0	0	1	2
	Did not receive both vaccinations	3	14	4	20	0	0	7	14

Supplemental Table 2. Summary of Solicited Adverse Events.

	HydroVax-001 1 mcg (N=20)			HydroVax-001 4 mcg (N=20)			Placebo (N=10)			All Subjects (N=50)		
**Solicited Event**	**n**	**%**	**95% CI**	**n**	**%**	**95% CI**	**n**	**%**	**95% CI**	**n**	**%**	**95% CI**
ANY Symptoms	15	75	51, 91	12	60	36, 81	6	60	26, 88	33	66	51, 79
Any Systemic Symptoms	11	55	32, 77	9	45	23, 68	6	60	26, 88	26	52	37, 66
Elevated Oral Temperature	0	0	0, 17	0	0	0, 17	0	0	0, 31	0	0	0, 7
Feverishness	3	15	3, 38	2	10	1, 32	2	20	3, 56	7	14	6, 27
Fatigue	7	35	15, 59	6	30	12, 54	4	40	12, 74	17	34	21, 49
Headache	6	30	12, 54	4	20	6, 44	5	50	19, 81	15	30	18, 45
Chills	2	10	1, 32	1	5	0, 25	1	10	0, 45	4	8	2, 19
Nausea	4	20	6, 44	2	10	1, 32	2	20	3, 56	8	16	7, 29
New muscle pain	1	5	0, 25	2	10	1, 32	1	10	0, 45	4	8	2, 19
Aggravated muscle pain	0	0	0, 17	0	0	0, 17	1	10	0, 45	1	2	0, 11
New joint pain	0	0	0, 17	1	5	0, 25	1	10	0, 45	2	4	0, 14
Aggravated joint pain	0	0	0, 17	0	0	0, 17	0	0	0, 31	0	0	0, 7
Any Local Symptoms	8	40	19, 64	11	55	32, 77	3	30	7, 65	22	44	30, 59
Pain at injection site	4	20	6, 44	5	25	9, 49	0	0	0, 31	9	18	9, 31
Tenderness	8	40	19, 64	9	45	23, 68	3	30	7, 65	20	40	26, 55
Induration/Swelling Measurement	0	0	0, 17	0	0	0, 17	0	0	0, 31	0	0	0, 7
Erythema/Redness Measurement	0	0	0, 17	0	0	0, 17	0	0	0, 31	0	0	0, 7
Induration/Swelling (functional grade)	2	10	1, 32	3	15	3, 38	1	10	0, 45	6	12	5, 24

N=Number of Subjects in the Safety population who received the specified dose.

95% Confidence Intervals are from an exact binomial distribution (Clopper-Pearson).

Supplemental Table 3. Summary of Solicited Adverse Events Post First Dose

	HydroVax-001 1 mcg (N=20)			HydroVax-001 4 mcg (N=20)			Placebo (N=10)			All Subjects (N=50)		
**Solicited Event**	**n**	**%**	**95% CI**	**n**	**%**	**95% CI**	**n**	**%**	**95% CI**	**n**	**%**	**95% CI**
ANY Symptoms	13	65	41, 85	10	50	27, 73	6	60	26, 88	29	58	43, 72
Any Systemic Symptoms	10	50	27, 73	6	30	12, 54	6	60	26, 88	22	44	30, 59
Elevated Oral Temperature	0	0	0, 17	0	0	0, 17	0	0	0, 31	0	0	0, 7
Feverishness	3	15	3, 38	1	5	0, 25	2	20	3, 56	6	12	5, 24
Fatigue	6	30	12, 54	5	25	9, 49	4	40	12, 74	15	30	18, 45
Headache	5	25	9, 49	2	10	1, 32	5	50	19, 81	12	24	13, 38
Chills	1	5	0, 25	1	5	0, 25	1	10	0, 45	3	6	1, 17
Nausea	3	15	3, 38	2	10	1, 32	1	10	0, 45	6	12	5, 24
New muscle pain	1	5	0, 25	1	5	0, 25	1	10	0, 45	3	6	1, 17
Aggravated muscle pain	0	0	0, 17	0	0	0, 17	1	10	0, 45	1	2	0, 11
New joint pain	0	0	0, 17	0	0	0, 17	1	10	0, 45	1	2	0, 11
Aggravated joint pain	0	0	0, 17	0	0	0, 17	0	0	0, 31	0	0	0, 7
Any Local Symptoms	6	30	12, 54	7	35	15, 59	2	20	3, 56	15	30	18, 45
Pain at injection site	3	15	3, 38	4	20	6, 44	0	0	0, 31	7	14	6, 27
Tenderness	6	30	12, 54	5	25	9, 49	2	20	3, 56	13	26	15, 40
Induration/Swelling Measurement	0	0	0, 17	0	0	0, 17	0	0	0, 31	0	0	0, 7
Erythema/Redness Measurement	0	0	0, 17	0	0	0, 17	0	0	0, 31	0	0	0, 7
Induration/Swelling (functional grade)	1	5	0, 25	1	5	0, 25	0	0	0, 31	2	4	0, 14

N=Number of Subjects in the Safety population who received the specified dose.
95% Confidence Intervals are from an exact binomial distribution (Clopper-Pearson).

Supplemental Table 4. Summary of Solicited Adverse Events Post Second Dose

	HydroVax-001 1 mcg (N=17)			HydroVax-001 4 mcg (N=16)			Placebo (N=10)			All Subjects (N=43)		
**Solicited Event**	**n**	**%**	**95% CI**	**n**	**%**	**95% CI**	**n**	**%**	**95% CI**	**n**	**%**	**95% CI**
ANY Symptoms	10	59	33, 82	8	50	25, 75	4	40	12, 74	22	51	35, 67
Any Systemic Symptoms	6	35	14, 62	4	25	7, 52	4	40	12, 74	14	33	19, 49
Elevated Oral Temperature	0	0	0, 20	0	0	0, 21	0	0	0, 31	0	0	0, 8
Feverishness	0	0	0, 20	1	6	0, 30	1	10	0, 45	2	5	1, 16
Fatigue	3	18	4, 43	1	6	0, 30	2	20	3, 56	6	14	5, 28
Headache	4	24	7, 50	3	19	4, 46	2	20	3, 56	9	21	10, 36
Chills	1	6	0, 29	0	0	0, 21	0	0	0, 31	1	2	0, 12
Nausea	2	12	1, 36	0	0	0, 21	1	10	0, 45	3	7	1, 19
New muscle pain	0	0	0, 20	1	6	0, 30	0	0	0, 31	1	2	0, 12
Aggravated muscle pain	0	0	0, 20	0	0	0, 21	0	0	0, 31	0	0	0, 8
New joint pain	0	0	0, 20	1	6	0, 30	1	10	0, 45	2	5	1, 16
Aggravated joint pain	0	0	0, 20	0	0	0, 21	0	0	0, 31	0	0	0, 8
Any Local Symptoms	5	29	10, 56	8	50	25, 75	1	10	0, 45	14	33	19, 49
Pain at injection site	1	6	0, 29	2	13	2, 38	0	0	0, 31	3	7	1, 19
Tenderness	4	24	7, 50	6	38	15, 65	1	10	0, 45	11	26	14, 41
Induration/Swelling Measurement	0	0	0, 20	0	0	0, 21	0	0	0, 31	0	0	0, 8
Erythema/Redness Measurement	0	0	0, 20	0	0	0, 21	0	0	0, 31	0	0	0, 8
Induration/Swelling (functional grade)	1	6	0, 29	2	13	2, 38	1	10	0, 45	4	9	3, 22

N=Number of Subjects in the Safety population who received the specified dose.
95% Confidence Intervals are from an exact binomial distribution (Clopper-Pearson

Supplemental Table 5. Standard $PRNT_{50}$ Seroresponse (4-Fold Rise), Geometric Mean Titer (GMT), and Geometric Mean Fold Rise (GMFR) by Study Day and Treatment Group, Modified Intent-To-Treat Population.

	HydroVax-001 1 mcg							HydroVax-001 4 mcg						
**Visit**	**N**	**4-Fold Rise**	**95% CI**	**GMT**	**95% CI**	**GMFR**	**95% CI**	**N**	**4-Fold Rise**	**95% CI**	**GMT**	**95% CI**	**GMFR**	**95% CI**
Baseline	20	-	-	5.0	-		-	19	-	-	5.0	-	-	-
Day 15 Post Dose 1	20	0 (0)	0, 17	5.0	-	1.0	-	19	0 (0)	0, 18	5.0	-	1.0	-
Day 29 Post Dose 1	20	0 (0)	0, 17	5.0	-	1.0	-	19	0 (0)	0, 18	5.0	-	1.0	-
Day 15 Post Dose 2	20	0 (0)	0, 17	5.0	-	1.0	-	18	5 (28)	10, 53	9.1	6.0, 13.7	1.8	1.2, 2.7
Day 29 Post Dose 2	20	0 (0)	0, 17	5.2	4.8, 5.6	1.0	1.0, 1.1	18	5 (28)	10, 53	9.4	5.4, 16.4	1.9	1.1, 3.3
Day 57 Post Dose 2	20	0 (0)	0, 17	5.0	-	1.0	-	17	2 (12)	1, 36	6.3	4.8, 8.1	1.3	1.0, 1.6
Day 180 Post Dose 2	20	0 (0)	0, 17	5.0	-	1.0	-	16	0 (0)	0, 21	5.0	-	1.0	-
Day 365 Post Dose 2	19	0 (0)	0, 18	5.0	-	1.0	-	16	0 (0)	0, 21	5.2	4.8, 5.7	1.0	1.0, 1.2

Supplemental Table 6. Complement-Enhanced $PRNT_{50}$ Seroresponse (4-Fold Rise), Geometric Mean Titer (GMT), and Geometric Mean Fold Rise (GMFR) by Study Day and Treatment Group, Modified Intent-To-Treat Population.

	HydroVax-001 1 mcg							HydroVax-001 4 mcg						
**Visit**	**N**	**4-Fold Rise**	**95% CI**	**GMT**	**95% CI**	**GMFR**	**95% CI**	**N**	**4-Fold Rise**	**95% CI**	**GMT**	**95% CI**	**GMFR**	**95% CI**
Baseline	20	-	-	5.0	-	-	-	19	-	-	5.0		-	-
Day 15 Post Dose 1	20	0 (0)	0, 17	5.0	-	1.0	-	19	1 (5)	0, 26	5.6	4.4, 7.0	1.1	0.9, 1.4
Day 29 Post Dose 1	20	0 (0)	0, 17	5.0	-	1.0	-	19	1 (5)	0, 26	5.6	4.4, 7.0	1.1	0.9, 1.4
Day 15 Post Dose 2	20	0 (0)	0, 17	5.0	-	1.0	-	18	8 (44)	22, 69	11.9	7.3, 19.4	2.4	1.5, 3.9
Day 29 Post Dose 2	20	0 (0)	0, 17	5.0	-	1.0	-	18	6 (33)	13, 59	10.4	6.7, 16.0	2.1	1.4, 3.2
Day 57 Post Dose 2	20	0 (0)	0, 17	5.0	-	1.0	-	17	2 (12)	1, 36	6.4	4.8, 8.5	1.3	1.0, 1.7
Day 180 Post Dose 2	20	0 (0)	0, 17	5.0	-	1.0	-	16	3 (19)	4, 46	6.5	4.8, 8.7	1.3	1.0, 1.8
Day 365 Post Dose 2	19	0 (0)	0, 18	5.0	-	1.0	-	16	2 (13)	2, 38	6.1	4.6, 8.1	1.2	0.9, 1.6

Supplemental Table 7. ELISA Seroresponse*Geometric Mean Titer (GMT) Results and Geometric Mean Fold Rise (GMFR) by Study Day and Treatment Group, Modified Intent-To-Treat Population.

	HydroVax-001							HydroVax-001						
	1 mcg							4 mcg						
**Visit**	**N**	**Seroconversion**	**95% CI**	**GMT**	**95% CI**	**GMFR**	**95% CI**	**N**	**Seroconversion**	**95% CI**	**GMT**	**95% CI**	**GMFR**	**95% CI**
Baseline	20	-	-	63.5	41.8, 96.7	-	-	19	-	-	43.1	34.6, 53.5	-	-
Day 15 Post Dose 1	20	3 (15)	3, 38	122.6	81.6, 184.2	1.9	1.5,2.5	19	2 (11)	1, 33	113.9	90.6, 143.2	2.7	2.1,3.4
Day 29 Post Dose 1	20	1 (5)	0, 25	115.8	75.7, 177.0	1.8	1.4,2.3	19	2 (11)	1, 33	118.7	95.2, 147.9	2.8	2.1,3.6
Day 15 Post Dose 2	20	7 (35)	15, 59	147.3	95.6, 226.9	2.3	1.7,3.2	18	12 (67)	41, 87	405.5	241.9, 679.8	9.1	5.2,15.8
Day 29 Post Dose 2	20	5 (25)	9, 49	142.9	90.2, 226.5	2.3	1.6,3.2	18	13 (72)	47, 90	401.5	249.9, 645.0	9.0	5.4,14.9
Day 57 Post Dose 2	20	5 (25)	9, 49	138.7	91.7, 209.7	2.2	1.7,2.9	17	11 (65)	38, 86	270.2	182.4, 400.5	5.9	3.8,9.3
Day 180 Post Dose 2	19	0 (0)	0, 18	62.7	39.9, 98.3	1.0	0.9,1.2	16	1 (6)	0, 30	68.2	50.0, 92.9	1.5	1.1,2.0
Day 365 Post Dose 2	19	1 (5)	0, 26	68.1	42.7, 108.6	1.0	0.8,1.2	16	1 (6)	0, 30	65.2	47.2, 89.9	1.4	1.2,1.8

*If a subject's baseline ELISA value was <200 and their follow-up visit ELISA value was >200, this was considered seroconversion. Alternatively, for subjects with a baseline ELISA value >200, the subject needed to demonstrate a four-fold rise at follow-up for seroconversion.
